# ModelTest-NG: A New and Scalable Tool for the Selection of DNA and Protein Evolutionary Models

**DOI:** 10.1093/molbev/msz189

**Published:** 2019-08-21

**Authors:** Diego Darriba, David Posada, Alexey M Kozlov, Alexandros Stamatakis, Benoit Morel, Tomas Flouri

**Affiliations:** 1 Computer Architecture Group, Centro de investigación CITIC, Universidade da Coruña, Elviña, A Coruña, Spain; 2 Computational Molecular Evolution Group, Heidelberg Institute for Theoretical Studies, Heidelberg, Germany; 3 Department of Biochemistry, Genetics, and Immunology, University of Vigo, Vigo, Spain; 4 Biomedical Research Center (CINBIO), University of Vigo, Vigo, Spain; 5 Galicia Sur Health Research Institute, Vigo, Spain; 6 Institute of Theoretical Informatics, Karlsruhe Institute of Technology, Karlsruhe, Germany; 7 Department of Genetics, Evolution and Environment, University College London, London, United Kingdom

**Keywords:** phylogenetic model selection, high-performance computing, efficient algorithms, phylogenetic inference

## Abstract

ModelTest-NG is a reimplementation from scratch of jModelTest and ProtTest, two popular tools for selecting the best-fit nucleotide and amino acid substitution models, respectively. ModelTest-NG is one to two orders of magnitude faster than jModelTest and ProtTest but equally accurate and introduces several new features, such as ascertainment bias correction, mixture, and free-rate models, or the automatic processing of single partitions. ModelTest-NG is available under a GNU GPL3 license at https://github.com/ddarriba/modeltest , last accessed September 2, 2019.

It is well known that the use of distinct probabilistic models of evolution can change the outcome of phylogenetic analyses (Buckley 2002; Buckley and Cunningham 2002; Lemmon and Moriarty 2004). Not surprisingly, a number of bioinformatic tools have been developed in the last 20 years for selecting the best-fit model for the data at hand (Posada and Crandall 1998; Posada 2008; Darriba et al. 2011, 2012; Kalyaanamoorthy et al. 2017). Although Abadi et al. (2019) concluded that using a parameter-rich model for DNA data leads to very similar inferences as the best-fit models, they average over a number of benchmark multiple sequence alignments (MSAs). However, looking at individual MSA analyses we may observe in some circumstances substantial topological differences between trees inferred under the best-fit model and under a parameter-rich GTR (Tavaré 1986) model (Arbiza et al. 2011; Hoff et al. 2016). Nowadays, continuous advances in sequencing technologies have made possible the assemblage of large MSAs that require faster and more scalable tools. In particular, our tools jModelTest (Darriba et al. 2012) and ProtTest (Darriba et al. 2011), which are among the most popular tools for DNA and protein model selection, despite implementing high-performance computing algorithms for parallel execution with dynamic load balancing, still rely on PhyML (Guindon and Gascuel 2003) for calculating the maximum likelihood (ML) scores for the competing models. This step constitutes the by far most compute-intensive part, requiring >99% of overall execution time. PhyML and hence jModelTest and ProtTest are relatively inefficient compared with more recent tools such as IQ-TREE (Nguyen et al. 2015). The model selection feature of IQ-TREE, called ModelFinder (Kalyaanamoorthy et al. 2017), is becoming increasingly popular due to its algorithmic and computational efficiency, the wide range of supported evolutionary models, and its user-friendliness. Another recently released tool for model selection is SMS (Smart Model Selection) (Lefort et al. 2017). SMS is based on PhyML and uses heuristic strategies to avoid evaluating the full set of candidate models.

Here, we introduce ModelTest-NG, a new program that outperforms its predecessors jModelTest and ProtTest in terms of speed. ModelTest-NG offers a completely redesigned graphical user interface and has several new capabilities. Its main features are as follows:

- *Data and models supported*: ModelTest-NG supports both nucleotide and amino acid models. It uses statistical criteria for selecting the best-fit substitution models such as AIC Akaike (1974), BIC Schwarz (1978), and DT Minin et al. (2003). It can select among all models included in jModelTest and ProtTest plus four other empirical amino acid replacement matrices and protein mixture models such as LG4M and LG4X (Le et al. 2012). ModelTest-NG can also assess the fit of a free-rate model (Yang 1995).
- *Partitioned MSAs*: ModelTest-NG can automatically perform model selection on single, nonoverlapping partitions, as specified by the user (e.g., on a per-gene basis, or by codon position).
- *Phylogenetic templates*: Users can select so-called templates for popular phylogenetic inference tools like RAxML (Stamatakis 2014), RAxML-NG (Kozlov et al. 2019), IQ-TREE, PhyML, PAUP (Swofford 2002), or MrBayes (Ronquist et al. 2012). When such a template is specified, ModelTest-NG will only evaluate models supported by the given tool and will print out the corresponding command line for phylogenetic reconstruction under the best-fit model.
- *Native implementation*: ModelTest-NG constitutes a full reimplementation of jModelTest and ProtTest in C++ that relies on a novel and efficient low-level implementation of the Phylogenetic Likelihood Library (PLL) (https://github.com/xflouris/libpll-2; last accessed September 2, 2019). This library encapsulates all compute- and memory-intensive phylogenetic likelihood computations and fully leverages the capabilities of modern x86 processors by using the AVX and AVX2 vector instruction sets. PLL also incorporates a recent algorithmic technique for accelerating likelihood calculations (Kobert et al. 2017). All required numerical optimization routines are implemented in the pll-modules library (https://github.com/ddarriba/pll-modules; last accessed September 2, 2019).
- *Parallel execution*: ModelTest-NG can take advantage of multicore desktop computers and clusters using *P*Threads and MPI (Message Passing Interface) (see supplementary material, Supplementary Material online for details).

We benchmarked ModelTest-NG against jModelTest, ProtTest, and ModelFinder (part of IQ-TREE version 1.6.1) using simulated as well as empirical data sets. We measured model selection accuracy (i.e., how often the generating model is recovered) using the simulated data sets, as well as run times. In all cases, we used the default model selection parameter settings. The experimental setup is described in detail in the supplementary material, Supplementary Material online, where we also further discuss the results.

ModelTest-NG found the true generating model for 81% of the simulated DNA MSAs (jModelTest: 81%, ModelFinder: 70%) and for 85% of the simulated protein MSAs (ProtTest: 85%, ModelFinder: 87%) (fig. 1). In general, the larger the data in terms of number of taxa and number of sites, the better ModelTest-NG performs compared with the competing tools (see fig. 1).

**Fig. 1:**
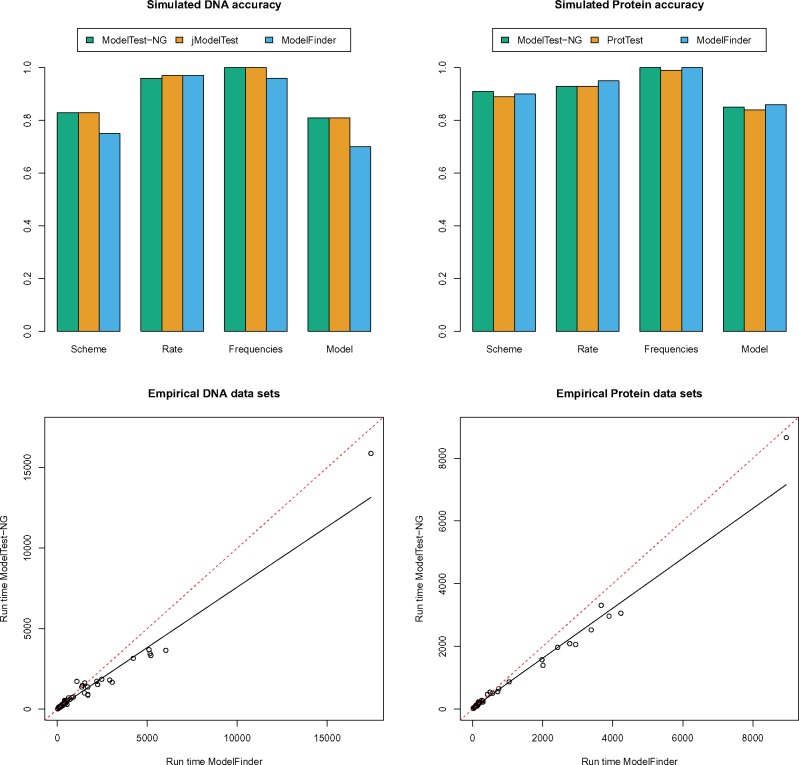
Model selection accuracy comparison between ModelTest-NG, jModelTest/ProtTest, and ModelFinder for simulated data (top) and LOESS curved fitted to a scatter plot of ModelTest-NG run times versus ModelFinder for empirical data (bottom), for DNA (left) and protein (right) MSAs. The dashed line represents equal run times.

In terms of speed, on simulated DNA data, ModelTest-NG was 110.77 times faster than jModelTest but slower than ModelFinder (the latter was 1.59 times faster). On empirical DNA data, ModelTest-NG yielded average speedups of 510.13 over jModelTest and of 1.24 over ModelFinder (supplementary fig. S1, Supplementary Material online). On simulated protein data, ModelTest-NG yielded average speedups of 36.07 over ProtTest, and similar run times as ModelFinder. On empirical protein data, ModelTest-NG was 36.94 times faster than ProtTest, and 1.19 times faster than ModelFinder. Importantly, ModelTest-NG seems to scale better than ModelFinder and jModelTest/ProtTest on large MSAs.

To ensure a fairer comparison with SMS, which only considers a subset of the models, we conducted a separate set of experiments comparing only ModelTest-NG and SMS on empirical data. For DNA data, both tools selected the same model 80% of the time, while ModelTest-NG was 95.53 times faster. For protein data, ModelTest-NG selected the same model as SMS 86.7% of the time and was 17.20 times faster.

The thoroughness of the model parameter optimization routines in ModelTest-NG can be controlled by the user. In additional experiments, we found that the more thoroughly we optimize the likelihood score the more accurate the selected model becomes (see Supplementary Material online). Possibly, the slight loss of accuracy in ModelFinder in our simulations can be explained by a less thorough default model optimization setting than in ModelTest-NG.

ModelTest-NG represents a substantial improvement over our previous tools, jModelTest and ProtTest. Although being equally accurate, it is up to two orders of magnitude faster on empirical data. Compared with ModelFinder, we observed similar run times for empirical data sets, but ModelFinder was faster on synthetic MSAs, particularly on DNA data. However, the accuracy of ModelFinder on DNA data was substantially lower than for ModelTest-NG (70% vs. 81%, respectively). In future versions of ModelTest-NG, we intend to introduce new methods to dynamically determine the optimal speed/accuracy tradeoff for the data set at hand. ModelTest-NG is particularly well suited for analyzing large data sets.

## Supplementary Material

msz189_Supplementary_Data
