# Supplementary material for: ModelTest-NG: A New and Scalable Tool for the Selection of DNA and Protein Evolutionary Models
Source: Mol Biol Evol. 2019 Aug 21;37(1):291–4. doi: 10.1093/molbev/msz189 (PMC6984357; doi:10.1093/molbev/msz189)
Supplement: msz189_Supplementary_Data [file msz189_supplementary_data.zip › msz189-Suppl_data/Supplementary_Material.pdf]

ModelTest-NG: a new and scalable tool for DNA and protein model selection

Darriba et al.

Supplementary Data

1. Test data sets

For the run time and accuracy assessments, we used five different collections empirical and simulated DNA and protein multiple sequence alignments (Table S1).

1. **Empirical RAxML DNA**. This is the empirical DNA data previously used for benchmarking RAxML (Stamatakis 2014) (available at https://github.com/stamatak/test-Datasets).
2. **Empirical IQ-TREE DNA.** This is the empirical DNA data used for benchmarking IQ-TREE (available at http://www.iqtree.org/ModelFinder/).
3. **Empirical IQ-TREE Protein.** This is the empirical protein data used for benchmarking IQ-TREE (available at http://www.iqtree.org/ModelFinder/).
4. **Simulated DNA.** This is the simulated DNA data generated in Darriba et al., 2012.
5. **Simulated Protein.** This is a simulated protein data set generated following the process described in Darriba et al., 2012.

Table S1: Summary of test data set collections. `Count` denotes the number of MSAs, `Taxa` is the number of sequences, `Sites` is the number of columns, and `Patterns` is the number of unique site patterns. Note that the actual number of unique site patterns determines the computational requirements, as identical sites are typically compressed into site patterns by all common phylogenetic inference tools.

		Count	Taxa			Sites			Patterns		
			Min	Avg	Max	Min	Avg	Max	Min	Avg	Max
DNA	Empirical RAxML	31	59	668	3782	297	2826	29198	234	1958	19437
	Empirical IQ-TREE	50	42	253	699	485	8428	93789	138	3129	32724
	Simulated	1000	10	55	100	501	999	1497	129	724	1491
PROT	Empirical IQ-TREE	45	26	81	194	127	2830	21155	108	2533	15022
	Simulated	1000	10	54	100	100	654	1200	46	481	1185

2. Evaluation setup

We benchmarked ModelTest-NG against the most popular model selection tools: jModelTest (Darriba et al., 2012), ProtTest (Darriba et al., 2011), ModelFinder (Kalyaanamoorthy et al., 2017), and SMS (Smart Model Selection) (LeFort et al., 2017). With the exception of SMS, the other tools deploy algorithms for selecting the best-fit model that are analogous to that of ModelTest-NG: they individually evaluate the score for each of the candidate models and use the same information criteria to select the best-fit model. In contrast, SMS uses a heuristic model search algorithm that reduces the number of models explored (see LeFort et al., 2017). For DNA data, SMS only selects among four different substitution models (GTR, TN93, HKY85, and K80) in contrast to the 22 substitution models that are available in ModelTest-NG by default. For protein data, SMS selects among 17 different empirical matrices and 2 among-site rate heterogeneity models (+G and +I+G). On average, SMS will evaluate the likelihood score for 6 to 8 DNA models and 30 protein models.

We divided the performance evaluation in 2 Sections: (1) a comparison against jModelTest, ProtTest, and ModelFinder; and (2) a comparison against SMS (Smart Model Selection). We present the evaluation of SMS separately, as it does not consider the same set of candidate models as the other tools. To compare ModelTest-NG with SMS in a fair way, we used a different ModelTest-NG configuration than in Section 2.1.

2.1 Exhaustive search

We compared ModelTest-NG version 0.1.5 with jModelTest version 2.1.10, ProtTest version 3.4.2 and ModelFinder version 1.6.1 (note that ModelFinder is the dedicated model selection module in IQ-TREE). For the sake of simplicity, all programs were executed in sequential execution mode using a single thread on a single physical core.

For DNA models, we used the GTR family of 88 nested models described in *Posada 2008*: 11 substitution schemes (JC/F81, K80/HKY, TrN, TPM1, TPM2, TPM3, TIM1, TIM2, TIM3, TVM, SYM/GTR) times four configurations of among-site rate variation (uniform, +I, +G, +I+G) times 2 stationary frequency models (equal or maximum-likelihood estimate). The command lines used were the following:

- `modeltest-ng -i <msa_file> -t mp -d nt`
- `iqtree -s <msa_file> -m TESTONLY -mfreq FO`
- `jModelTest.jar -d <msa_file> -t BIONJ -S 11 -i -f -g4 -BIC -AIC -AICc -DT -tr 1`

For protein models, we compared the 144 models available by default in ProtTest and in ModelFinder version 1.6.1: 18 empirical substitution matrices times four configurations of among-site rate variation (uniform, +I, +G, +I+G) times two stationary frequencies (fixed or

empirical). The STMTREV model was excluded from the ModelTest-NG analysis as it was neither included in ModelFinder nor in ProtTest. For real data sets, we also included the LG4X and LG4M mixture models. The command lines we used were the following:

- modeltest-ng -i <msa_file> -t mp -d aa -m -STMTREV,+LG4M,+LG4X
- iqtree -s <msa_file> -st AA -m TESTONLY -madd LG4X,LG4M
- ProtTest.jar -i <msa_file> -I -G -IG -F -BIC -AIC -AICC -DT -S 0 -threads 1"

To evaluate performance we measured the run times. The execution times for ModelTest-NG, jModelTest, and ProtTest correspond to the overall wall clock time. Note that ModelFinder also optimizes model parameters for the selected best-fit model after the model selection process. Therefore, to conduct a fair comparison, we only measured the time required by ModelFinder for the actual model selection process.

We measure speedups via two distinct metrics: “local average” is the average speedup over all individual per-MSA speedups, and “global” is the overall speedup for analyzing all MSAs in the respective test data set (i.e., the ratio of accumulated run times for evaluating the entire test data set). This global speedup value better reflects the potential computational savings since a speedup of say 1.2 will yield substantially larger CPU resource savings on a large MSA that takes hours to analyze than on a small MSA that requires a few seconds to run.

For each test data set collection, we also compared the respective model selection results: best-fit model, substitution scheme, among-site rate variation model and nucleotide/amino acid frequencies.

2.2 SMS Heuristic Search

We compared ModelTest-NG version 0.1.5 with SMS (Smart Model Selection) version 1.8.1. As mentioned above, SMS uses heuristic strategies that significantly reduce the runtime compared to an exhaustive model search. In addition, SMS implements a smaller set of candidate models than ModelTest-NG.

For DNA models, SMS selects among a set of 16 candidate substitution models: 4 substitution matrices (K80, HKY, TrN, GTR) times 4 configurations of among-site rate variation (uniform, +I, +G, +I+G). In addition to the configuration described in the previous Subsection, we executed ModelTest-NG with the smallest possible set of models that contains the 16 candidate models assessed by SMS. The command lines used were the following:

- modeltest-ng -i <msa_file> -t mp -d nt -s 3
- sms.sh -i <msa_file> -d nt -c BIC

For protein models, SMS supports 68 amino-acid replacement models: 17 empirical matrices times 2 configurations of among-site rate variation (+G, +I+G) times two stationary frequencies types (fixed by the model or empirical from the input data). In addition to the configuration described in the previous Subsection, we executed ModelTest-NG with the +G and +I+G models, only.

- modeltest-ng -i <msa_file> -t mp -d aa -h gf
- sms.sh -i <msa_file> -d aa -c BIC

We measure speedups via the two metrics introduced in Section 2.1 (i.e., “local average” and “global” speedup). We also compared the respective model selection results (best-fit model, substitution scheme, among-site rate variation model, stationary frequencies). For this test, we only used the empirical data sets because they comprise substantially larger MSAs, and hence justify the use of a heuristic model search algorithm.

3. Computing infrastructure used

We executed all single-node benchmarks on an Intel i7-2600 system with 4 physical cores, equipped with 16GB RAM and swapping disabled (see Table S2 for details).

Table S2: Technical data of the computing cluster used for our sequential and parallel experiments using pthreads.

Hardware		Software	
CPU model	Intel i7-2600	OS	Ubuntu 16.04.5 LTS
CPU Architecture	Sandy Bridge		
Cores	4 @ 3.40GHz	Compiler	Gcc 5.2.0
Memory	16GB DDR3		
Vector Extensions	AVX, SSE4		

For the MPI parallel benchmark, we used a cluster of 15 identical nodes, each one with the features described in Table S3.

Table S3: Technical data of the computing cluster used for our parallel experiments using MPI.

Hardware		Software	
CPU model	2xIntel Xeon E5-2660	OS	Rocks 6.1
CPU Architecture	Sandy Bridge		
Cores	16 @ 2.20GHz	Compiler	Gcc 5.2.0
Memory	64GB DDR3		
Vector Extensions	AVX, SSE4		

4. Results

4.1 Speed

Table S4 below shows the speedups between ModelTest-NG and the competing tools for all test data sets.

Our evaluations show that ModelTest-NG is on average 1 to 2 orders of magnitude faster than both jModelTest, and ProtTest. In particular, for large MSAs in terms of the number of sequences/taxa (e.g., MSAs included in the DNA RAxML test data set collection), differences in run times were even more pronounced. For some MSAs in the **Empirical RAxML DNA** test data set collection, we observed speedups exceeding a factor of 3,000.

Figure S1 shows the run times. Compared to ModelFinder, ModelTest-NG exhibited slightly better run times for the empirical DNA test data sets (average speedup of 1.16 and 1.08 for the RAxML and ModelFinder test data sets, respectively). For the empirical protein test data set, the average speedup was 1.00. The global speedup was generally better than the local average speedup, indicating that ModelTest-NG scales better than ModelFinder on larger data sets (Figure S2).

Table S4: Local and global speedups between ModelTest-NG and competing tools. "Local Average" is the average speedup over all individual per-MSA speedups, and "Global" is the overall speedup for analyzing all MSAs in the respective test data set (i.e., ratio of accumulated run times for evaluating the entire test data set)

	ProtTest/jModelTest		ModelFinder	
	Local Average	Global	Local Average	Global
Simulated DNA	116.23	110.77	0.63	0.63
Empirical DNA RAxML	369.83	855.39	1.16	1.25
Empirical DNA IQ-TREE	193.23	164.87	1.08	1.24
Simulated Protein	36.79	36.07	0.47	1.03
Empirical Protein IQ-TREE	36.94	42.31	1.00	1.19

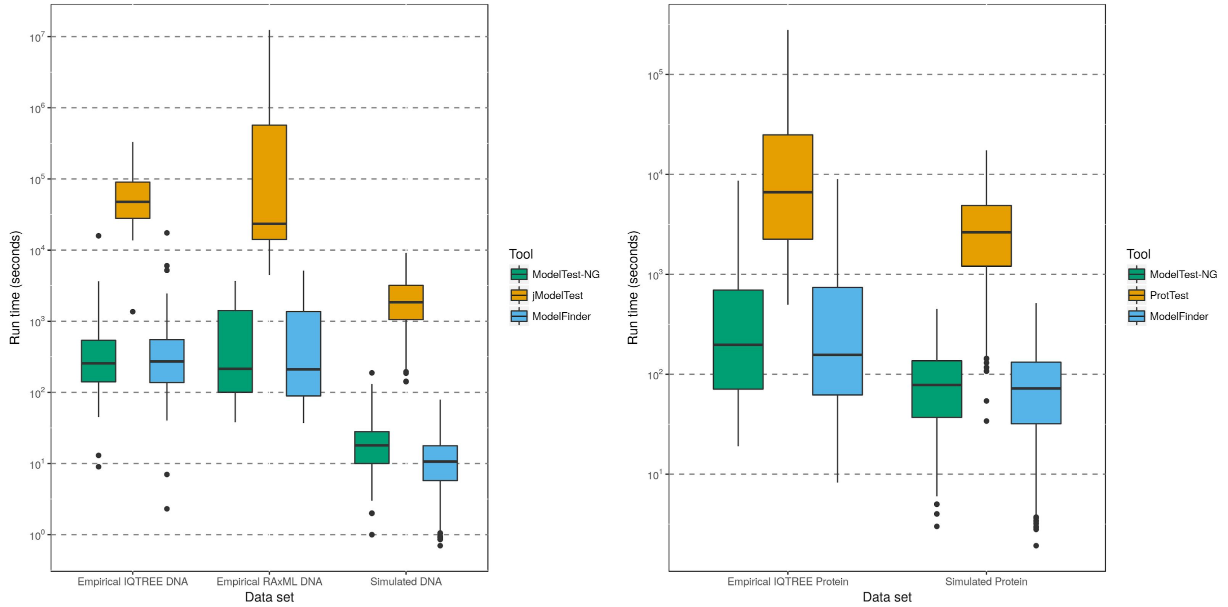

Figure S1: Run time comparison between ModelTest-NG, jModelTest/ProtTest, and ModelFinder*, for empirical and simulated DNA (left) and protein (right) MSAs.Note the logarithmic scale on the 'y' axis.*

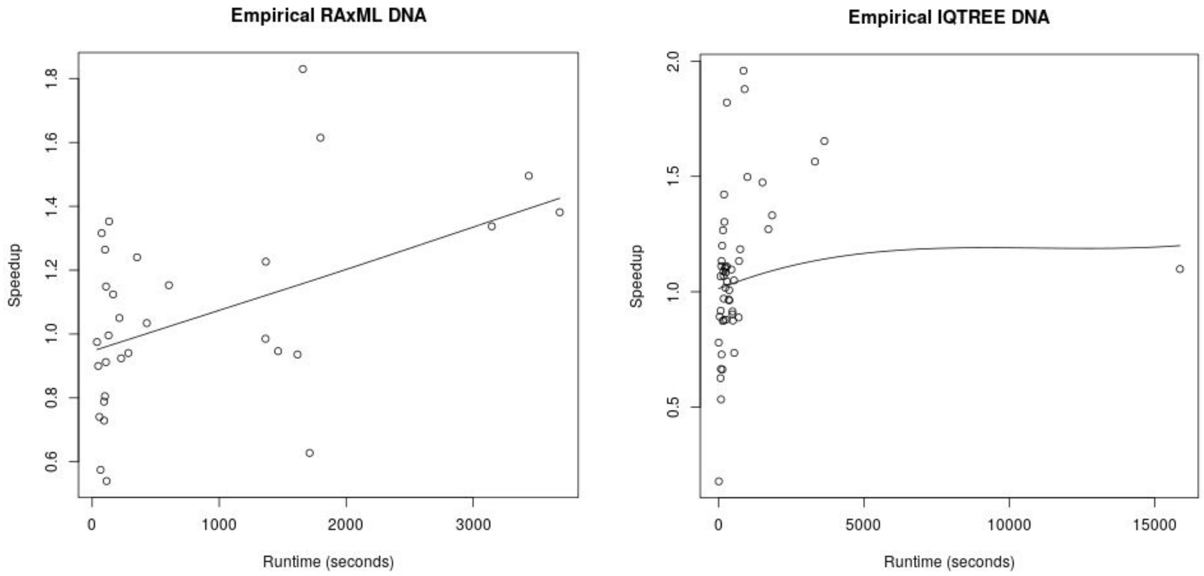

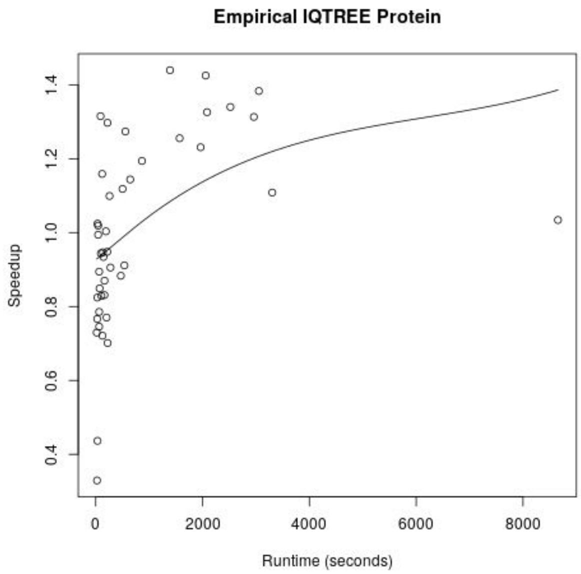

Figure S2: LOESS curve fitted to a scatter plot of ModelTest-NG speedups versus ModelFinder according to the ModelTest-NG run times for empirical data sets.

Figure S3 shows the run times for the experiment described in Section 2.2. For DNA data, ModelTest-NG was on average 68.84 times faster than SMS, with a global speedup of 95.53. For protein data, ModelTest-NG was on average 14.17 times faster than SMS, with a global speedup of 17.20. Moreover, even though the candidate models assessed by ModelTest-NG were set up such as to contain those evaluated by SMS, SMS performs a heuristic search and does not evaluate every single candidate model in contrast to ModelTest-NG.

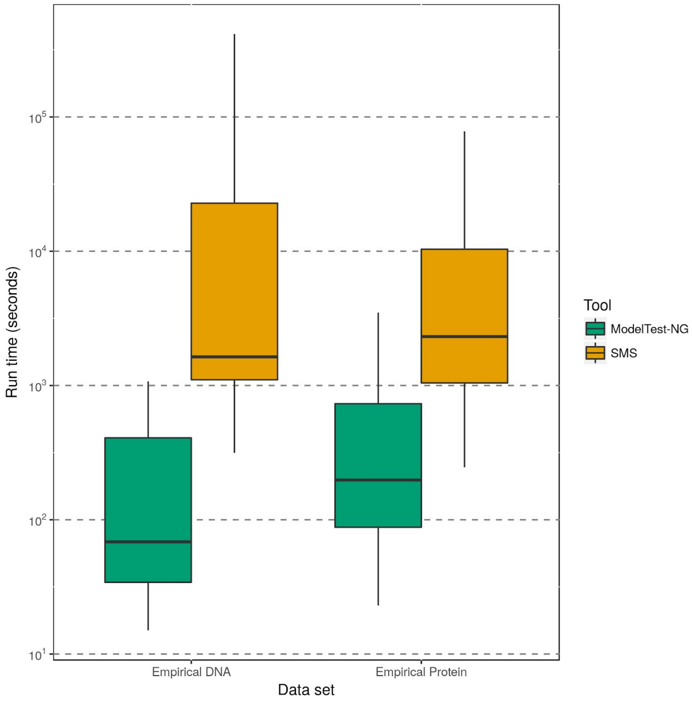

Figure S3: Run time comparison between ModelTest-NG and SMS for empirical DNA and protein data. Note the logarithmic scale on the 'y' axis.

4.2 *Accuracy*

In our comparison of the selected best-fit models on simulated data against the known ground truth (Table S5 and Figure S4), ModelTest-NG shows analogous results to jModelTest and ProtTest, identifying the true generating model for 81% and 84% of the simulated DNA and protein MSAs, respectively. ModelFinder performed worse (70%), while for protein data its performance was analogous to ModelTest-NG as well as ProtTest (also 84%).

If we compare the results obtained by the different tools for the RAxML test data set collection (Table S5), we observe that ModelTest-NG and jModelTest attain 100% agreement with respect to the selected substitution scheme and among site rate variation model. ModelTest-NG and ModelFinder agree on the best-fit model for 90% of the MSAs.

For DNA data, ModelTest-NG and SMS attain a 100% agreement with respect to the selected among site rate variation model, and 80% on the best-fit model. However, note that even though we configured ModelTest-NG to match SMS, the candidate models set available in SMS is still a strict subset of the candidate models in ModelTest-NG. For protein data, ModelTest-NG and SMS agree on the best-fit empirical substitution matrix and stationary frequencies model for 100% of the MSAs. However, they agree on the exact best-fit model for 86.7% of the MSAs (i.e., the remaining 13.3% differ on the among site rate variation model).

4.3 Discussion

Overall, ModelTest-NG is slightly faster than ModelFinder on empirical datasets, whereas on simulated data it is slightly slower. However, for simulated DNA data, the increased speed of ModelFinder comes at the cost of a slight decrease in accuracy. The general trend is that ModelTest-NG shows higher speedups with increasing MSA size, both in terms of number of taxa and MSA patterns.

The SMS heuristic yields the same best-fit model as ModelTest-NG for most datasets. However, ModelTest-NG is substantially faster. In addition, optimizing the complete set of candidate models as ModelTest-NG does, can provide a more comprehensive understanding of model selection results. We can, for instance, calculate the weight of each model according to appropriate Information Criteria and derive a quantitative measurement of confidence in the selection results.

Table S5. Model selection results comparison against the `ground truth` (for simulated data sets) and against ModelTest-NG. Values show the fraction by which the models selected by the tools agree. `Model` is the overall substitution model (i.e., combination of `Scheme`, `Rate` and `Freqs`). `Scheme` is the substitution scheme (DNA) or empirical substitution matrix (protein data). `Rate` is the among site rate variation model . `Freqs` is the stationary frequency type (equal or ML estimate for DNA data sets; defined by the model or empirical for protein data sets).

			**Accuracy**							
			**Vs Ground Truth**				**MTNG vs …**			
			**Model**	**Scheme**	**Rate**	**Freqs**	**Model**	**Scheme**	**Rate**	**Freqs**
**DNA**	**SIM**	**ModelTest-NG**	0.81	0.83	0.96	1.00	-	-	-	-
		**jModelTest**	0.81	0.84	0.97	1.00	0.97	0.99	0.98	1.00
		**ModelFinder**	0.70	0.75	0.97	0.97	0.83	0.89	0.98	0.96
	**RML**	**ModelTest-NG**	-	-	-	-	-	-	-	-
		**jModelTest**	-	-	-	-	0.87	1.00	1.00	0.87
		**ModelFinder**	-	-	-	-	0.90	0.94	1.00	0.97
	**IQT**	**ModelTest-NG**	-	-	-	-	-	-	-	-
		**jModelTest**	-	-	-	-	0.91	0.94	1.00	0.94
		**ModelFinder**	-	-	-	-	0.94	0.94	1.00	1.00
**AA**	**SIM**	**ModelTest-NG**	0.85	0.90	0.93	1.00	-	-	-	-
		**ProtTest**	0.85	0.90	0.93	0.99	0.94	0.97	0.96	0.99
		**ModelFinder**	0.87	0.90	0.95	1.00	0.97	0.99	0.98	1.00
	**IQT**	**ModelTest-NG**	-	-	-	-	-	-	-	-
		**ProtTest**	-	-	-	-	0.91	0.98	0.93	1.00
		**ModelFinder**	-	-	-	-	0.89	0.93	0.96	1.00

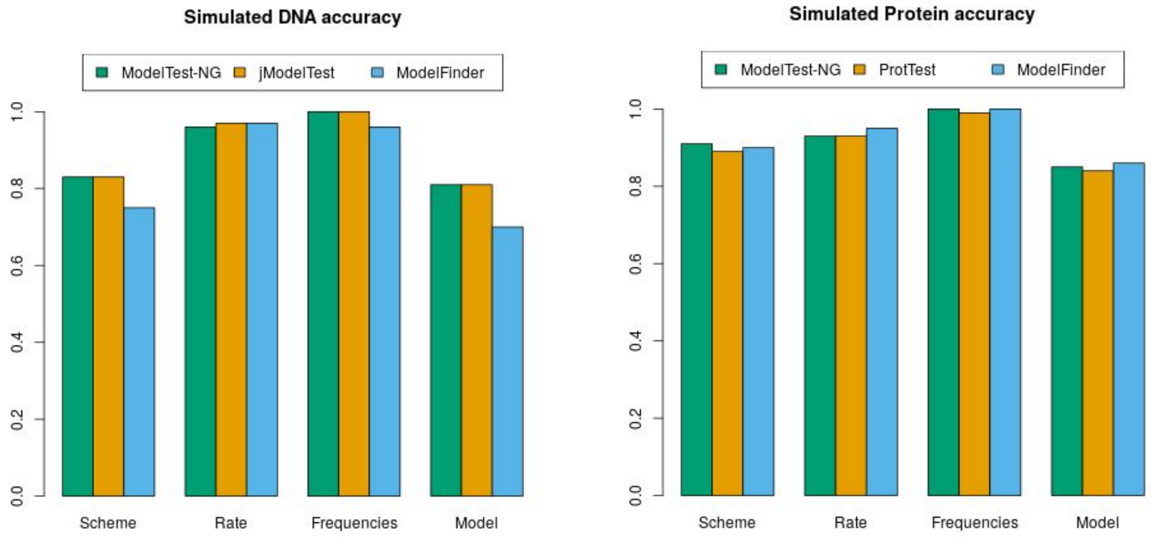

Figure S4. Model selection accuracy results. Comparison against the `ground truth` (for simulated data sets). "Scheme" is the substitution scheme (i.e., rate matrix symmetries for DNA data and empirical matrix for protein data), "Rate" is the configurations of among-site rate variation, "Frequencies" is the stationary frequencies model, and "Model" is the combination of the previous 3 parameters.

5. Model Optimization Thoroughness.

ModelTest-NG optimizes model parameters with a tolerance of 0.01 log-Likelihood units by default. This tolerance provides a ‘good’ trade-off between selection accuracy and speed. To further investigate the impact of this value on execution times, we re-analyzed the simulated DNA MSAs using distinct tolerance settings. We used tolerances of 0.01, 0.1, and 0.5 log-likelihood units, and the observed model inference accuracy was 81%, 76%, and 66% respectively. The model inference accuracy was calculated as the fraction of samples where the true generating model was found. We observe that, if we increase this tolerance setting, both, execution time, and accuracy decreases (Table S6 and Figure S5).

Table S6. Model selection results comparison against the `ground truth` (for simulated data sets) for ModelTest-NG, according to the model parameter optimization tolerance (in log-Likelihood units). Values show the fraction by which the models selected by the tools agree. `Model` is the overall substitution model (i.e., combination of `Scheme`, `Rate` and `Freqs`). `Scheme` is the substitution scheme (DNA) or empirical substitution matrix (protein data). `Rate` is the among site rate variation model . `Freqs` is the stationary frequency type (equal or ML estimate for DNA data sets; defined by the model or empirical for protein data sets). `Run time` is the average run time among the MSAs in the data set.

Tolerance	Model	Scheme	Rate	Freqs	Run time
**0.01**	0.805	0.834	0.961	1.000	20.690
**0.10**	0.764	0.821	0.925	1.000	20.140
**0.50**	0.660	0.751	0.880	0.995	16.890

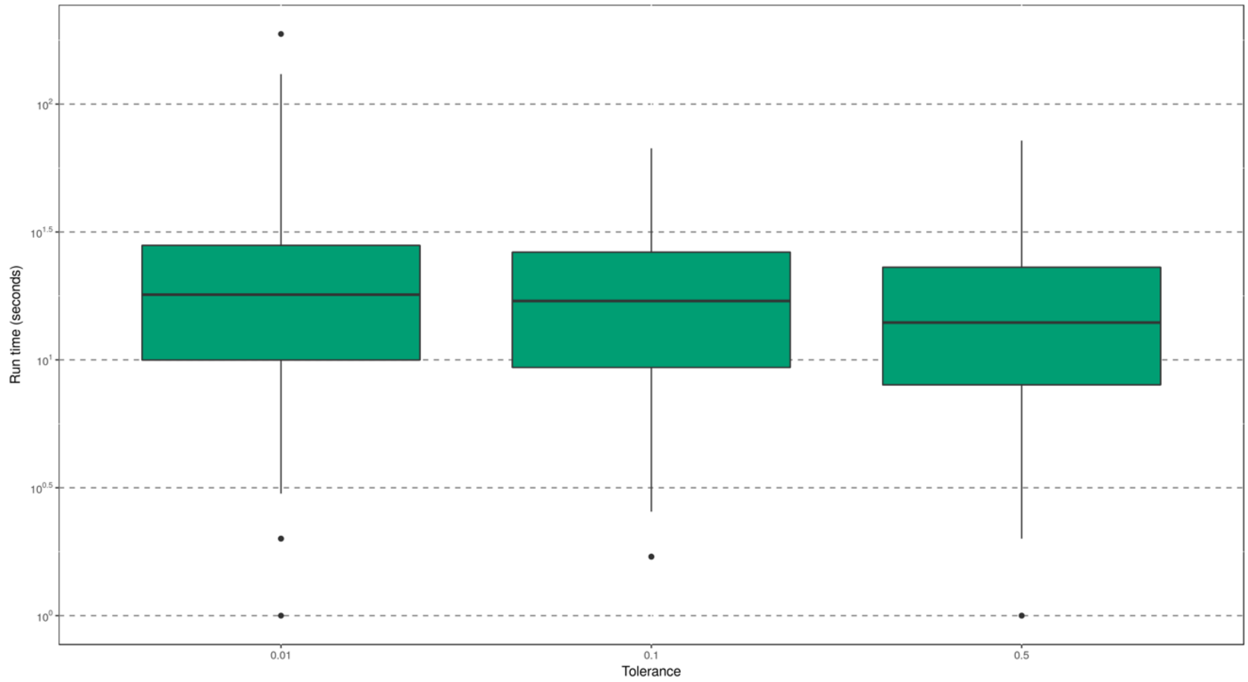

Figure S5. ModelTest-NG run times according to the model optimization tolerance (in log-Likelihood units). Note the logarithmic scale on the y-axis.

6. Coarse-grained parallelization with MPI and PThreads

The ModelTest-NG strategy for model selection consists in optimizing model parameters and branch length for each of the candidate models. In addition to vector level parallelism, ModelTest-NG also offers coarse-grained task-level parallelism in two flavors: PThreads for shared memory systems, and MPI (Message Passing Interface) for distributed memory architectures.

For vector level parallelism, ModelTest-NG will automatically detect the best set of vector instructions available on the processor (AVX2 or SSE3), and use the respective computational kernels to achieve optimal performance. The task-level parallel strategy consists in distributing the distinct independent model optimization tasks among the available processors.

By default, ModelTest-NG will execute a single thread, but it will issue a warning if there are more resources available on the system (i.e., if there is more than one physical core available). The reason for using one thread as default is that ModelTest-NG (as of v0.1.5) is optimized for sequential execution. It accelerates model parameters and branch lengths optimizations by reusing information from the previously assessed models. Hence, the initial candidate models usually require more time for parameter optimization than the subsequent ones. When ModelTest-NG is executed in parallel, it starts by optimizing several models at the same time without this prior information it re-uses. Consequently, this decreases parallel efficiency.

We separately tested the parallel performance of using *PThreads* on a single multi-core node, and of MPI on a compute cluster. For the *PThreads* version, the command line used was the following:

- `modeltest-ng -i <msa_file> -d <nt/aa> -p <number_of_threads>`

For MPI version, the command line used was the following:

- `mpirun -n <number_of_processes> modeltest-mpi -i <msa_file> -d <nt/aa>`

Figure S6 shows the speedups obtained with pthreads and MPI on the hardware systems described in Section 3 (Table S2 for PThreads and Table S3 for MPI). We evaluated the PThreads version with up to 4 threads on a computer with 4 physical cores.

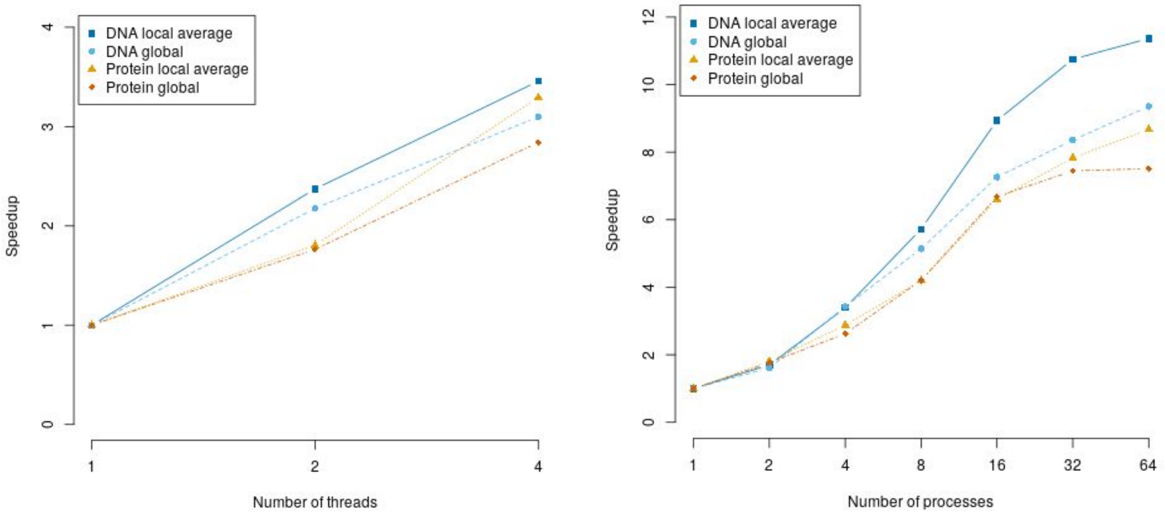

Figure S6: Average speedups of ModelTest-NG using pthreads (left) and MPI (right).

References

- Darriba, D., Taboada, G. L., Doallo, R., & Posada, D. (2011). ProtTest 3: fast selection of best-fit models of protein evolution. Bioinformatics, 27(8), 1164-1165.
- Darriba, D., Taboada, G.L., Doallo, R., and Posada, D.2012. jmodeltest 2: more models, new heuristics and parallel computing. Nature methods, 9(8): 772–772
- Gascuel O. 1997. BIONJ: an improved version of the NJ algorithm based on a simple model of sequence data.MolBiolEvol.14(7):685–695.
- Kalyaanamoorthy, S., Minh, B. Q., Wong, T. K., von Haeseler, A., & Jermiin, L. S. (2017). ModelFinder: fast model selection for accurate phylogenetic estimates. *Nature methods*, *14*(6), 587.
- Lefort V, Longueville JE, Gascuel O. SMS: Smart Model Selection in PhyML. Mol Biol Evol. 2017;34(9):2422–4. 10.1093/molbev/msx149
